# Concurrent use of Chinese herbal medicine and anticoagulants may reduce major bleeding events

**DOI:** 10.1371/journal.pone.0271965

**Published:** 2022-08-23

**Authors:** Shuo-Min Hsu, Hung-Jen Lin, Yi-Wei Kao, Te-Mao Li, Ben-Chang Shia, Sheng-Teng Huang

**Affiliations:** 1 School of Chinese Medicine, China Medical University, Taichung, Taiwan; 2 Department of Chinese Medicine, China Medical University Hospital, Taichung, Taiwan; 3 Graduate Institute of Business Administration, Fu Jen Catholic University, Taipei, Taiwan; 4 Research Cancer Center for Traditional Chinese Medicine, Department of Medical Research, China Medical University Hospital, Taichung, Taiwan; 5 An-Nan Hospital, China Medical University, Tainan, Taiwan; National University of Kaohsiung, TAIWAN

## Abstract

**Background:**

This retrospective cohort study investigated the risk of major bleeding events during the concurrent use of Chinese herbal medicine (CHM) and anticoagulants in clinical practice.

**Methods:**

A total of 4,470 patients receiving anticoagulant drugs were selected from Taiwan’s National Health Insurance Research Database (NHIRD). Half (n = 2,235) were also using CHMs (CHM cohort); the other half were not (non-CHM cohort). Each cohort was matched 1:1 using the propensity score. Chi-square testing and the Student’s *t*-test were used to examine differences between two cohorts. Cox proportional hazard regression analysis assessed the risks for major bleeding events in each cohort, as well as bleeding risks associated with specific CHM formulas and herbs. Cumulative incidence curves for major bleeding events were calculated using Kaplan-Meier analysis.

**Results:**

Compared with the non-CHM cohort, the CHM cohort had a lower risk of overall bleeding events (p < 0.001) including hemorrhagic stroke (p = 0.008), gastrointestinal (GI) bleeding (p < 0.001), urogenital bleeding (p ≤ 0.001) and nasal/ear/eye bleeding (p = 0.004). Single herbs, such as *Glycyrrhiza uralensis et Rhizoma*, *Panax notoginseng*, *Panax ginseng*, *Platycodon grandiflorum*, *Eucommia ulmoides Oliver* and formulas, such as Shu Jing Huo Xue Tang, Shao Yao Gan Cao Tang and Ji Sheng Shen Qi Wan were associated with a lower risk of major bleeding events.

**Conclusion:**

Using CHMs with anticoagulants appeared to decrease the risk of major bleeding, especially CHMs products containing *Glycyrrhiza uralensis et Rhizoma*, *Panax notoginseng*, *Panax ginseng*, *Platycodon grandiflorum* and *Eucommia ulmoides Oliver*. Further investigations are needed to determine whether CHM can maintain the therapeutic efficacy of anticoagulants while simultaneously reducing potential side effects.

## Introduction

Cardiovascular and cerebrovascular diseases are primary causes of death worldwide, accounting for 32% of all deaths reported in 2019 [1]. Antiplatelet and anticoagulant therapies are widely prescribed for the prevention of death from these diseases [2] and many of these patients are likely to combine their prescriptions with complementary and alternative medicines (CAMs) [3, 4]. For instance, an analysis of data on CAM use among 10,572 respondents in the 2002 United States National Health Interview Survey found that over one-third of those with cardiovascular disease had used CAMs in the previous 12 months while receiving cardiac medications [3]. Of patients visited to CAM providers, 38.1% was reported risk factors for cardiovascular disease and 11.4% had a diagnosis of cardiovascular disease within the population of US adults [5].

Among the different types of CAM, Chinese medicine has the most potential of achieving universal health coverage under support of the World Health Organization [6]. However, compared with other cardiovascular drugs, anticoagulant agents are associated with a greater risk of potentially harmful interactions, when co-administered with herbal medicines [7]. Importantly, the concomitant use of Chinese medicines and anticoagulants has been found to alter the metabolism and action of anticoagulants such as warfarin, necessitating an adjustment to their doses to ensure patient safety and therapeutic efficacy [8]. Besides, although fewer in number than warfarin, herb-drug interactions in direct oral anticoagulants are likely to remain unnoticed until a complication occurs since INR are not routinely monitored [9].

The mechanisms underlying herb–drug interactions remain an understudied area of pharmacotherapy due to variability in herbal product composition, uncertainty of the causative constituents, and often scant knowledge of causative constituent pharmacokinetics [10]. Moreover, Western herbs involved in clinical herb-warfarin interactions have been much better classified than interactions involving Chinese herbal medicines (CHMs) and warfarin [8]. These limitations mean that the majority of physicians and professional trainees have limited training on herbal adverse events, toxicities, and drug interactions [11]. This lack of training and education may result in insufficient recognition of potential herb-drug interactions, specifically their bleeding occurrences and associated adverse events [11]. Considering the potential occurrences of serious interactions and their undesired consequences, researchers have proposed that the concomitant use of CHMs and Western medications should be continuously investigated [12].

Randomized controlled trials (RCTs) have been widely recognized as the best means of research for evaluating the efficacy and safety of medical interventions. Although small RCTs involving single herbs in herb-anticoagulant interactions have been performed [13], prescriptions of traditional Chinese medicines (TCMs) most commonly involve formulas, which are not easy to incorporate into RCT designs [14]. The characteristics of TCM mean that decoctions cater to individual differences, making it difficult to evaluate clinical efficacy of formulas in population-based RCTs [15]. In cases such as the above clinical situations, which are unlikely to be examined with RCTs, observational studies are particularly valuable and may provide valid and reliable real-world evidence [16]. Thus, a larger sample size from a retrospective cohort study might provide a holistic view of clinical evidence for herb-drug reactions.

To guarantee the safety and efficacy of CHMs in patients already receiving anticoagulant drugs, we conducted this retrospective population-based cohort study to compare age, sex, prevalence of comorbidities and drug treatments among patients registered in Taiwan’s National Health Insurance Research Database (NHIRD). We aimed to examine transient events associated with hospitalization due to major bleeding events in patients receiving anticoagulant drugs, with or without CHMs. Further analysis of the CHM cohort included specific CHM herbs which might cause major bleeding events.

## Material and methods

### Data source

Data originated from the 2005 Longitudinal National Health Insurance Database (LHID2005) provided by the Health and Welfare Statistics Application Center, Ministry of Health and Welfare, Taiwan. The NHI Program was launched in Taiwan in March 1995 and since 2010, has enrolled more than 99% of the inhabitants of Taiwan [17].

The LHID2005 contains all the original claims data for a randomly selected sample of one million beneficiaries enrolled in the NHI program in 2005. Analysis of the NHI data using the multistage stratified systematic sampling method has established that there are no statistically significant differences regarding sex or age between these one million insured individuals and the general population. The LHID2005 contains all NHI enrolment files, claims data and the registry for prescription drugs, providing comprehensive information on the beneficiaries from 1996 to 2013. Reimbursed TCM services, including Chinese herbal products, acupuncture/moxibustion, and Chinese traumatology therapy in ambulatory clinics, are also included in the database.

### Ethical approval

To protect the privacy of patients, Taiwan’s National Health Research Institute (NHRI) has encrypted the names of patients, health care providers and medical institutions, to permit public access for research purposes [17]. Thus, the Review Board waived the requirement for obtaining informed consent from the patients. This study was conducted in accordance with the Declaration of Helsinki and was approved by the Institutional Review Board of China Medical University, Taichung, Taiwan (CMUH-104-REC2-115-R4).

### Study population and variables

**Fig 1:**
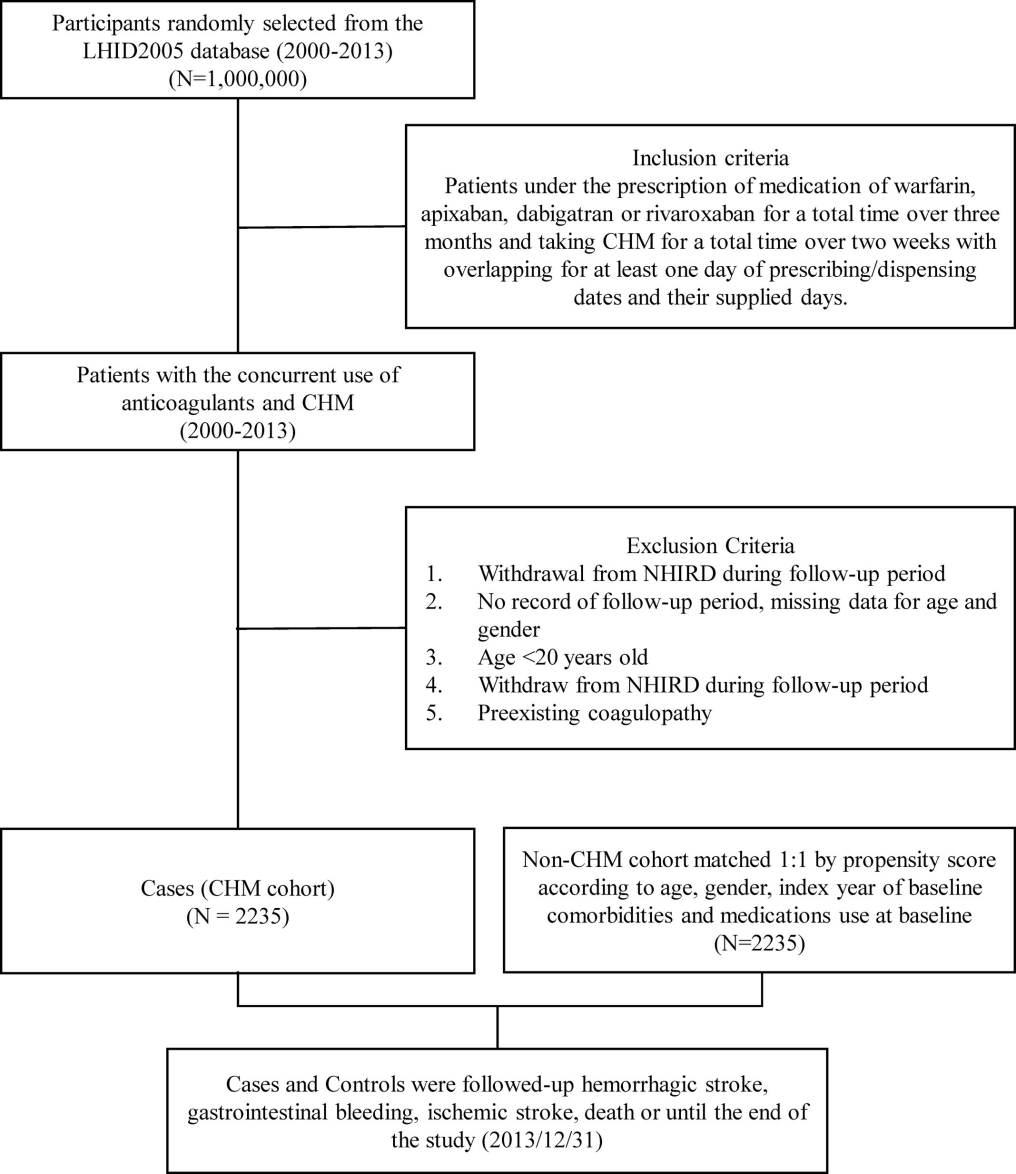
Flow diagram of the study enrollment process.

To investigate the safety and efficacy of the concurrent use of CHM and anticoagulants, we identified two cohorts: one using both CHMs and anticoagulants (CHM cohort) and a cohort using only anticoagulants (non-CHM cohort) as shown in Fig 1.

The inclusion criteria for the CHM cohort specified that patients were prescribed anticoagulants consisting of warfarin, apixaban, dabigatran, or rivaroxaban for at least one continuous week and were concomitantly using CHM for at least one continuous week with an overlap of at least 1 day in prescribing/dispensing dates and their supplied days between January 2000 and December 2013. Patients diagnosed with preexisting coagulopathy (ICD-9-CM 286, 287), were aged <20 years, or who withdrew from the NHIRD during the follow-up period were excluded in both cohorts (Fig 1).

The non-CHM cohort included patients using anticoagulants consisting of warfarin, apixaban, dabigatran, or rivaroxaban for at least one continuous week, without any CHM prescriptions.

Patients in the non-CHM cohort were matched 1:1 by propensity score with the CHM cohort according to age, gender, index year of baseline comorbidities and medication use at baseline. Age and the baseline comorbidities were adjusted according to the CHA2DS2–VASc score, which is used to assess stroke and bleeding risks in patients with atrial fibrillation [18]. We separated the age with three ranges from 20 to 64, 65 to 74 and over 75 years old respectively due to increasing risk of stroke and thromboembolism defined by the CHA2DS2–VASc score [18]. Also, comorbidities which might affect drug metabolism such as chronic kidney disease and abnormal liver function were taken into consideration. We defined a patient with a history of comorbidities as having been diagnosed at least once in the Emergency Department (ED) or Inpatient Department before the index date for diabetes (ICD-9-CM 250), hypertension (ICD-9-CM 401, 405), chronic kidney disease (ICD-9-CM 580–589, A350), previous ischemic stroke/transient ischemic attack (TIA)/thromboembolism (TE) (ICD-9-CM 433, 434, 435, 437, 438, 452, 453), myocardial infarction (MI)/peripheral arterial disease (PAD)/aortic plaque (ICD-9-CM 410, 411, 412, 413, 414, 440, 444, 445), abnormal liver function (ICD-9-CM 070, 570, 571, 572, 573), or heart failure (ICD-9-CM 402, 404). Due to different mechanisms and drug-drug interactions of warfarin and direct oral anticoagulants, anticoagulants with warfarin, apixaban, dabigatran and rivaroxaban were also adjusted. Other baseline medications included aspirin and antiplatelet agents, as shown in S1 Table in S1 File.

The primary outcome included hemorrhagic stroke (ICD-9-CM 430, 431, 432), GI bleeding (ICD-9-CM 530, 531, 532, 534, 533, 534, 535, 537, 562, 569, 578, 784.8, 786) and urogenital bleeding (ICD-9-CM 596, 599, 602, 608, 620, 621, 623, 625, 626, 627, 640, 641, 666, 674), as well as nose and eye bleeding (ICD-9-CM 360, 362, 364, 372, 374, 376, 379, 386, 388, 784) confirmed by diagnosis once in the ED or during hospitalization within the study period. For all study participants, the end date of follow-up was whichever came first: (1) from the first day of prescribing/dispensing dates of CHMs or anticoagulants, up until 8 weeks after the last prescribing/dispensing dates of CHMs or anticoagulants; (2) withdrawal from the database; or (3) the occurrence of a major bleeding event (Fig 1).

### Statistical analysis

After matching both cohorts with the propensity score to reduce confounding bias, the Chi-square test was used to assess differences between the CHM and non-CHM cohorts in categorical variables, represented by numbers and percentages. Cox proportional hazard regression associated with hazard ratios (HRs), adjusted hazard ratios (aHRs) and 95% confidence intervals (CIs) was used to assess between-cohort risks for major bleeding events. Incidence rates per 10,000 person-years were estimated for both cohorts. The Kaplan-Meier method was used to estimate cumulative incidence rates of bleeding. The log-rank test was applied to compare the difference between two bleeding incidence curves. We also analyzed CHMs previously reported as possibly involved in herb-drug interactions with anticoagulants and we identified the top 10 single herbs and formulas most frequently used in the CHM cohort, to investigate potential associations with major bleeding events. Cox proportional hazard regression analysis was used to study further associations of major bleeding events with particular herbs or formulas, and calculated the adjusted hazard ratios (aHRs) and 95% confidence intervals (CIs) for the cumulative risk of survival.

Data analysis was performed using SAS version 9.4 (SAS Institute Inc., Cary, NC, USA). Values for the cumulative incidence curve were plotted by R software (R Foundation for Statistical Computing, version 3.6.1, Vienna, Austria http://www.r-project.org). A *p*-value of less than 0.05 in a two-sided test was considered statistically significant.

## Results

2,235 patients met the criteria of the CHM cohort while 8,031 patients met the criteria of the non-CHM cohort. After matching 1:1 by propensity score with the CHM cohort, a total of 4,470 subjects were enrolled in the database: 2,235 patients in the CHM cohort and 2,235 in the non-CHM cohort. In the total study population, numbers of females and males were almost equal. Most of the study participants were aged from 20 to 64 years.

Compared with the non-CHM cohort, there were slightly more patients with heart failure in the CHM cohort. Other comorbidities in the CHM cohort included hypertension (48.2%), diabetes mellitus (22.0%), chronic kidney disease (6.1%), previous ischemic stroke/TIA (42.1%), MI/PAD or aortic plaque (42.2%), abnormal liver function (13.2%), and heart failure (4.4%) (Table 1). None of these comorbidities differed significantly between the CHM and non-CHM cohorts, except for heart failure (Table 1).

**Table 1:** Demographic characteristics, comorbidities and medications of patients using anticoagulants in Taiwan during 2000–2013.

	CHM cohort	Non-CHM cohort	p value
Total N = 4470	2235	2235	
**SEX = Male (%)**	1179 (52.8)	1199 (53.6)	0.569
**Age at baseline (%)**			0.973
20–64	1152 (51.5)	1148 (51.4)	
65–74	647 (28.9)	654 (29.3)	
≥75	436 (19.5)	433 (19.4)	
**Baseline comorbidity** = Yes			
Hypertension (%)	1078 (48.2)	1083 (48.5)	0.905
Chronic kidney disease (%)	137 (6.1)	129 (5.8)	0.658
Diabetes mellitus (%)	491 (22.0)	491 (22.0)	1.000
Previous ischemic stroke/TIA /TE (%)	940 (42.1)	934 (41.8)	0.880
MI/ PAD/aortic plaque (%)	944 (42.2)	948 (42.4)	0.928
Abnormal liver function (%)	296 (13.2)	280 (12.5)	0.503
Heart failure (%)	98 (4.4)	71 (3.2)	0.041
**Baseline Medications** = Yes			
Warfarin (%)	2212 (99.0)	2213 (99.0)	1.000
Dabigatran & Rivaroxaban (%)	23 (1.0)	22 (1.0)	1.000
Aspirin (%)	1050 (47.0)	1048 (46.9)	0.976
Other antiplatelet agents (%)	790 (35.3)	783 (35.0)	0.851

Abbreviations: TIA, transient ischemic attack; TE, thromboembolism; MI, Myocardial infarction; PAD, peripheral arterial disease.

Among patients in the CHM cohort, 99.0% were taking warfarin, with rest of the 1.0% taking dabigatran or rivaroxaban. Almost half (47%) of the patients in CHM cohort were using aspirin; 35.3% were using other antiplatelet agents. Medications did not differ significantly between the cohorts.

**Fig 2:**
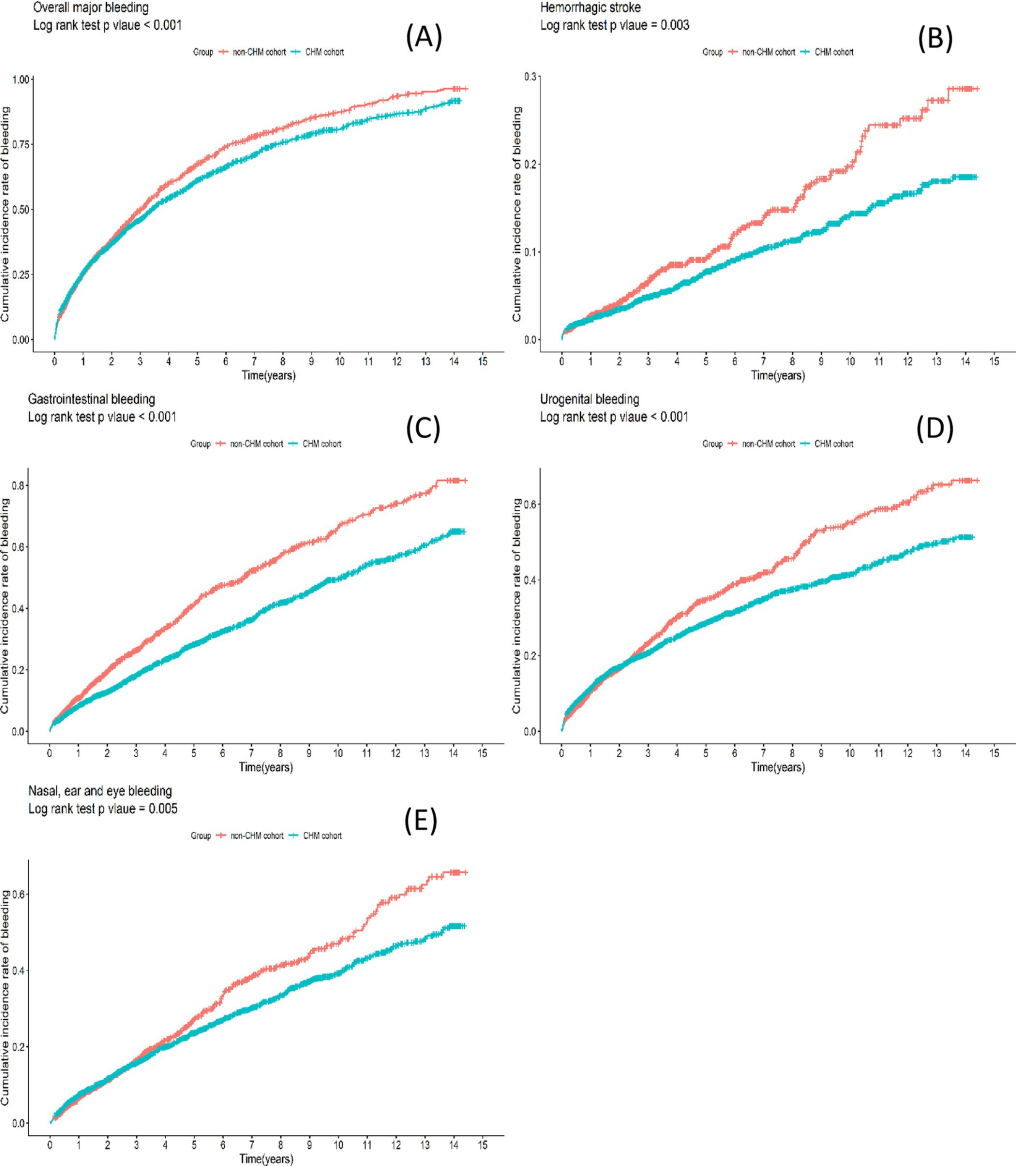
Kaplan–Meier analysis showing cumulative incidence of major bleeding events included (A) overall bleeding, (B) hemorrhagic stroke, (C) gastrointestinal bleeding, (D) urogenital bleeding and (E) nasal, ear, and eye bleeding for both CHM and non-CHM cohorts during a 15-year follow-up.

From the first day of anticoagulant or CHM administration up until 8 weeks after the last prescribing/dispensing dates, 2,623 patients were hospitalized due to major bleeding events: 1,439 (19.69%) in the CHM cohort and 1,184 (23.86%) in the non-CHM cohort. Most patients in the CHM cohort (n = 753, 6.94%) were hospitalized for GI bleeding, followed by urogenital bleeding (n = 655, 6.49%), nasal/ear/eye bleeding (n = 589, 5.37%), or hemorrhagic stroke (n = 195, 1.56%). The risk of overall bleeding events was lower in the CHM cohort than in the non-CHM cohort (aHR = 0.873, 95% CI = 0.808~0.943, p < 0.001), as was hemorrhagic stroke (aHR = 0.742, 95% CI = 0.594~0.926, p < 0.008), GI bleeding (aHR = 0.653, 95% CI = 0.587~0.727, p < 0.001), urogenital bleeding (aHR = 0.824, 95% CI = 0.732~0.928, p < 0.001) and nasal/ear/eye bleeding (aHR = 0.822, 95% CI = 0.720~0.938, p < 0.004) (Table 2). Kaplan-Meier analysis revealed that the CHM cohort had a significantly lower cumulative incidence of overall bleeding, hemorrhagic stroke, GI, urogenital, and nasal/ear/eye bleeding (Fig 2).

**Table 2:** Incidence rates, hazard ratios and confidence intervals of different major bleeding events between CHM and non-CHM cohort.

	CHM cohort	Non- CHM cohort	CHM cohort incidence rate*	Non-CHM cohort incidence rate*	HR 95C.I.	HR p value	aHR† 95C.I.	aHR p value
Overall bleeding	1439	1184	1969.523	2386.941	0.870(0.805~0.940)	<0.001	0.873(0.808~0.943)	<0.001
Hemorrhagic stroke	195	138	156.634	236.485	0.715(0.573~0.892)	0.003	0.742(0.594~0.926)	0.008
Gastrointestinal bleeding	753	646	694.299	1103.046	0.641(0.576~0.713)	<0.001	0.653(0.587~0.727)	<0.001
Urogenital bleeding	655	493	649.174	879.851	0.814(0.724~0.916)	<0.001	0.824(0.732~0.928)	0.001
Nasal, ear, and eye bleeding	589	370	537.706	669.754	0.827(0.725~0.943)	0.005	0.822(0.720~0.938)	0.004

Abbreviations: IR, incidence rates, HR, hazard ratio; aHR, adjusted hazard ratio; CI, confidence interval.

* Incidence rate: per 10,000 person-years.

†Adjusted HR: adjusted for age, gender, comorbidities, and medications in Cox proportional hazards regression.

We analyzed bleeding events associated with the single herbs that have previously exhibited the potential to interact with anticoagulants [19, 20]. *Glycyrrhiza uralensis et Rhizoma* (liquorice root) (aHR = 0.854, 95% CI = 0.739~0.986, p = 0.05), *Panax notoginseng* (notoginseng) (aHR = 0.837, 95% CI = 0.731~0.959, p = 0.05) and *Panax ginseng* (Asian ginseng) (aHR = 0.762, 95% CI = 0.596~0.974, p = 0.05) were found to have a lower risk of major bleeding events. No statistically significant differences were observed between the remaining seven herbs as shown in Table 3. Thus, the data from this study indicate that these 10 herbs were not associated with higher risks of bleeding when co-administered with anticoagulants based on this large retrospective cohort study.

**Table 3:** Statistics of CHMs previously reported for possible herb-drug interactions with anticoagulants with frequency of prescription, average duration for prescription, average daily dose and hazard ratio hazard ratio of major bleeding events between CHM and non-CHM cohort.

N = 2,235	Frequency of prescription, N (%)	Average duration for prescription (days)	Average daily dose (g)	HR(95C.I.)	aHR(95C.I.) †
*Salvia miltiorrhiza*	684 (30.6)	14.45 (64.64)	7.40 (66.75)	0.929 (0.834, 1.035)	0.948 (0.851, 1.056)
*Glycyrrhiza uralensis*	326 (14.6)	4.47 (31.24)	3.35 (54.34)	0.843 (0.730, 0.973)*	0.854 (0.739, 0.986)*
*Zingiber officinale*	185 (8.3)	2.33 (18.77)	1.67 (28.42)	0.865 (0.721, 1.039)	0.893 (0.742, 1.074)
*Levisticum officinale*	37 (1.7)	0.50 (6.58)	0.15 (1.54)	1.168 (0.791, 1.723)	1.212 (0.820, 1.793)
*Carthamus tinctorius*	39 (1.7)	0.56 (9.24)	0.17 (1.68)	1.118 (0.782, 1.598)	1.186 (0.828, 1.699)
*Curcuma longa*	221 (9.9)	3.10 (22.16)	1.69 (31.86)	0.933 (0.788, 1.104)	0.966 (0.815, 1.144)
*Prunus persica*	140 (6.3)	1.66 (12.24)	1.14 (18.41)	0.889 (0.723, 1.094)	0.922 (0.748, 1.136)
*Panax notoginseng*	384 (17.2)	5.47 (33.16)	3.62 (54.41)	0.822 (0.718, 0.941)*	0.837 (0.731, 0.959)*
*Ginkgo biloba*	165 (7.4)	1.59 (10.54)	0.62 (2.76)	0.891 (0.736, 1.078)	0.919 (0.759, 1.114)
*Panax ginseng*	102 (4.6)	1.43 (15.35)	2.68 (51.03)	0.729 (0.571, 0.930)*	0.762 (0.596, 0.974)*

Abbreviations: HR, hazard ratio; aHR, adjusted hazard ratio; CI, confidence interval.

* p < 0.05

†aHR: adjusted for age, gender, comorbidities, and medications in Cox proportional hazards regression.

An analysis of the top 10 single herbs and formulas prescribed by TCM practitioners revealed that *Salvia miltiorrhiza* (danshen) was the most frequently used single herb and Shu Jing Huo Xue Tang the most frequently prescribed formula. When combined with anticoagulants, none of the single herbs or formulas had any significant effects upon bleeding events. *Platycodon grandiflorum* (balloon flower root) (aHR = 0.850, 95% CI = 0.744~0.970, p < 0.05), *Eucommia ulmoides Oliver* (Eucommia bark) (aHR = 0.840, 95% CI = 0.728~0.968, p < 0.05), *Glycyrrhiza uralensis et Rhizoma* (liquorice root) (aHR = 0.854, 95% CI = 0.739~0.986, p < 0.05) significantly lowered the risk of major bleeding events compared with anticoagulants alone. The formula Shu Jing Huo Xue Tang (n = 582, 26.0%) was also associated with a significantly lower risk of major bleeding events when compared with anticoagulants alone (aHR = 0. 871, 95% CI = 0. 778~0. 975, p < 0.05). Moreover, the formulas Shao Yao Gan Cao Tang (aHR = 0.844, 95% CI = 0.744~0.958, p < 0.05) and Ji Sheng Shen Qi Wan (aHR = 0.866, 95% CI = 0.755~0.994, p < 0.05) lowered the risk of major bleeding events in adjusted analyses (Table 4). Herbal components included in the top 10 formulas are listed in S2 Table in S1 File.

**Table 4:** Statistics of the top 10 prescribed CHMs with frequency of prescription, average duration for prescription, average daily dose and hazard ratio of major bleeding events between CHM and non-CHM cohort.

N = 2,235	Frequency of prescription, N (%)	Average duration for prescription (days)	Average daily dose (g)	HR(95C.I.)	aHR(95C.I.)†
Single herbs					
*Salvia miltiorrhiza*	684 (30.6)	14.45 (64.64)	7.40 (66.75)	0.929 (0.834, 1.035)	0.948 (0.851, 1.056)
*Corydalis yanhusuo*	488 (21.8)	5.43 (26.83)	3.68 (35.32)	0.887 (0.786, 1.001)	0.894 (0.791, 1.011)
*Platycodon grandiflorum*	406 (18.2)	5.08 (23.31)	3.93 (41.22)	0.844 (0.739, 0.963)*	0.850 (0.744, 0.970)*
*Fritillariae thunbergii*	389 (17.4)	4.77 (20.81)	4.79 (60.84)	0.915 (0.801, 1.046)	0.927 (0.811, 1.060)
*Scutellaria baicalensis*	368 (16.5)	4.82 (23.29)	4.07 (44.98)	0.982 (0.859, 1.122)	1.022 (0.894, 1.169)
*Astragalus membranaceus*	368 (16.5)	6.87 (42.42)	2.96 (30.22)	0.986 (0.863, 1.127)	0.998 (0.873, 1.142)
*Puerariae lobatae*	353 (15.8)	3.81 (20.99)	3.52 (44.36)	0.895 (0.780, 1.029)	0.896 (0.780, 1.030)
*Eucommia ulmoides*	327 (14.6)	4.48 (26.86)	2.78 (47.51)	0.838 (0.728, 0.966)*	0.840 (0.728, 0.968)*
*Ophiopogon japonicus*	327 (14.6)	5.09 (39.17)	3.26 (49.97)	0.885 (0.770, 1.019)	0.901 (0.782, 1.037)
*Glycyrrhiza uralensis*	326 (14.6)	4.47 (31.24)	3.35 (54.34)	0.843 (0.730, 0.973)*	0.854 (0.739, 0.986)*
Formulas					
Shu Jing Huo Xue Tang.	582 (26.0)	5.98 (28.03)	15.85 (142.46)	0.868 (0.776, 0.972)*	0.871 (0.778, 0.975)*
Zhi Gan Cao Tang.	540 (24.2)	11.37 (53.36)	16.19 (141.06)	0.884 (0.785, 0.996)*	0.906 (0.803, 1.021)
Shao Yao Gan Cao Tang.	448 (20.0)	4.17 (19.47)	11.92 (137.12)	0.843 (0.744, 0.956)*	0.844 (0.744, 0.958)*
Xue Fu Zhu Yu Tan.	429 (19.2)	6.23 (34.74)	18.01 (189.11)	0.863 (0.759, 0.981)*	0.907 (0.797, 1.032)
Du Huo Ji Sheng Tan.	411 (18.4)	5.48 (26.47)	17.62 (195.46)	0.940 (0.827, 1.067)	0.939 (0.827, 1.067)
Ji Sheng Shen Qi Wan.	373 (16.7)	6.92 (36.17)	12.70 (136.64)	0.875 (0.764, 1.003)	0.866 (0.755, 0.994)*
Ge Gen Tang.	359 (16.1)	3.30 (19.05)	10.60 (103.40)	0.885 (0.772, 1.016)	0.903 (0.786, 1.038)
Sheng Mai Yin.	346 (15.5)	5.74 (32.17)	8.07 (93.40)	0.884 (0.769, 1.016)	0.895 (0.778, 1.029)
Liu Wei Di Huang Wan.	339 (15.2)	4.25 (19.85)	18.25 (269.90)	0.980 (0.854, 1.123)	0.980 (0.854, 1.125)
Ping Wei San.	338 (15.1)	4.12 (27.70)	12.39 (168.06)	0.894 (0.778, 1.028)	0.917 (0.797, 1.055)

Abbreviations: HR, hazard ratio; aHR, adjusted hazard ratio; CI, confidence interval.

* p < 0.05

†aHR: adjusted for age, gender, comorbidities, and medications in Cox proportional hazards regression.

## Discussion

In this retrospective cohort study, the overall incidence of any major bleeding events was significantly lower among patients using CHMs combined with anticoagulants compared with those using anticoagulants alone. The evidence also suggests that the use of some CHMs may significantly decrease the risk of major bleeding events. To the best of our knowledge, this is the first retrospective cohort study to show a negative correlation between the prescription of CHMs with anticoagulants and major bleeding events.

In analyses adjusted for baseline values and other covariates, the occurrence of any major bleeding event was significantly lower in the CHM cohort compared with the non-CHM cohort. Among all major bleeding events, GI bleeding had the highest incidence rate in both cohorts, which corresponds with a previous study [21]. However, although GI bleeding is the most common side effect of anticoagulants, the risk of mortality is relatively lower with GI bleeding (6%) compared with intracranial bleeding (38%) [21]. In our study, the risks of both GI bleeding and intracranial bleeding were reduced in the CHM cohort to approximately to one-third of these bleeds occurring in the non-CHM cohort.

Our analysis of the prescribed CHMs in this study found that CHM herbs previously involved in possible herb-drug interactions with anticoagulants did not increase the incidence of major bleeding events. Moreover, *Glycyrrhiza uralensis* (licorice root), *Panax notoginseng* (notoginseng root), *Panax ginseng* (Asian ginseng), *Platycodon grandiflorum* (balloon flower root) and *Eucommia ulmoides* (Eucommia bark) reduced the risk of major bleeding events. Similarly, the formulas of Shu Jing Huo Xue Tang, Shao Yao Gan Cao Tang and Ji Sheng Shen Qi Wan were associated with a significantly lower hazard ratio of major bleeding events. We therefore reviewed those previous studies in an attempt to determine the possible reasons for bleeding tendencies with combined Eastern and Western medicines.

*Glycyrrhiza uralensis* (licorice root) has been frequently used not only in CHM but also as a Western folk remedy throughout history, and commonly as an additional sweetener in foods. One case report described how an 80-year-old woman prescribed warfarin for atrial fibrillation developed black tarry stools and an elevated international normalized ratio (INR) after consuming a pound of licorice [22]. As a CYP3A4, CYP2B6 and CYP2C9 inhibitor, licorice might decrease warfarin metabolism and therefore increase the INR [23]. An antiplatelet effect has also been reported with licorice [24]. Paradoxically, licorice also activates the pregnane X receptor and increases warfarin clearance in rats [25]. The *Glycyrrhiza uralensis* used for CHM is usually honey-roasted [26], which differs from the crude *Glycyrrhiza uralensis* used in animal studies. Thus, the controversies surrounding the use of *Glycyrrhiza uralensis* do not relate to its use in TCM practice and therefore need further validation.

No clinical studies or case reports have described interactions between notoginseng and warfarin, although experimental studies in rats have found that administration of notoginseng total saponins for 5 weeks increases the time to reach peak concentration (t_max_) half-life and the warfarin area under the curve [19]. Notoginseng possesses both pro-hemostatic and anti-hemostatic properties. Different notoginseng total saponins may either increase or decrease platelet aggregation in the same experimental setting. Further clinical studies are therefore needed to study the mechanism in patients co-administered notoginseng and anticoagulants [27].

Contradictory results have been reported in small-scale RCTs in regard to probable interactions between warfarin and *Panax ginseng*, due to different dosage administrations [28]. Ginseng has been associated with some episodes of spontaneous bleeding, but also with reports of subtherapeutic INR and thrombosis in patients previously stable on warfarin [19]. Regardless of the fact that there are different presumed mechanisms underlying the interaction between warfarin and ginseng with consequently different outcomes [28], we recommend that experts be consulted before the concomitant use of ginseng extracts and warfarin.

As a traditional herbal medicine, *Platycodon grandiflorus* has been widely used in Northeast Asia (including China, Japan, and Korea) for the treatment of bronchitis, asthma, pulmonary tuberculosis, diabetes, tonsillitis, and inflammatory diseases [29, 30]. Although animal experiments have shown that *Platycodon grandiflorum* saponins inhibits platelet aggregation and therefore may act as a potential anticoagulant [29, 30], no clinical reports exist of herb-drug interactions between *Platycodon grandiflorum* and anticoagulants.

Hypertension has often been linked to bleeding risk and, in particular, may elevate the risk of intracranial hemorrhage [31]. *Eucommia ulmoides* is a high-quality, natural antihypertensive drug without side effects during long-term use [32]. *In vitro* and *in vivo* experiments have revealed that its antihypertensive effects are achieved by promoting nitric oxide (NO) release and inhibiting the production of angiotensin converting enzyme (ACE) and angiotensin (Ang II) [33]. This antihypertensive feature of *Eucommia ulmoides* may explain the lower incidence of major bleeding events when used with anticoagulants.

Animal experiments have revealed that the concurrent use of Shu Jing Huo Xue Tang and warfarin may potentiate bleeding events by increasing prothrombin time (PT) and activated partial thromboplastin time (APTT) [34, 35]. However, no further evidence is available from clinical studies or case reports on adverse effects of the concurrent use of Shu Jing Huo Xue Tang and anticoagulants. In contrast to data from animal models, our real-world data demonstrate that using Shu Jing Huo Xue Tang with anticoagulants ameliorates bleeding events.

Shao-Yao-Gan-Cao-Tang, a traditional herbal medicine with a blend of 50% *Paeoniae radix* and 50% *Glycyrrhiza uralensis*, has been used for a very long time in TCM and is known to be effective for relieving cramp-related pain in GI smooth muscle and skeletal muscle [36, 37]. Although no clinical reports exist of herb-drug interactions for Shao Yan Gan Cao and anticoagulants, *Glycyrrhiza uralensis* is one of the major components in Shao Yan Gan Cao and might contribute to the lower incidence of major bleeding events observed in our study.

Ji Sheng Shen Qi Wan inhibits enhanced platelet aggregation in diabetic rats [38] and a clinical study has reported vasodilatory action due to the increased production of NO [39]. However, no reports exist of interactions between Ji Sheng Shen Qi Wan and anticoagulants.

Our study reveals that in clinical practice, patient with the concurrent use of CHM had lower incidence rate of major bleeding events during antithrombotic therapy. Regardless of previous data concerning specific CHMs with contradictory results to our evidence, animal experiments are less likely to explain real-world practice, as such experiments cannot consider complicated clinical conditions and individualized therapies in TCM. The complexity of clinical interventions and evaluations in TCM means that it is virtually impossible to conduct an RCT with a sufficiently large sample and long-term evaluation [40]. Thus, our study is the most likely means of representing results from TCM practice in patients with anticoagulants.

While the concurrent use of CHM and anticoagulants can lower the risk of major bleeding events, dosing was limited in this study. On the other hand, interactions between CHMs and anticoagulants may increase the chance of thrombosis. So far, one clinical report also based on NHIRD has stated that CHM users had a lower risk of new-onset ischemic stroke and less likelihood of hospitalization within 1 year compared with non-CHM users [41]. Thus, it is possible to have fewer bleeding events on anticoagulants while maintaining their therapeutic effects and prevent bleeding tendencies, if they are used properly in combination with CHMs.

Several limitations in this study need to be addressed. Firstly, we only included anticoagulant users who had been hospitalized or admitted to the ED due to major bleeding events. We did not evaluate patients who suffered from mild or moderate bleeding and did not require hospitalization (i.e., they were treated in outpatient clinics). Secondly, only concentrated Chinese herbal products prescribed by TCM physicians are reimbursed by Taiwan’s NHI program and are thus recorded in the available databases. The NHIRD databases do not include raw Chinese herbs or dietary supplement products. Moreover, the NHIRD does not provide detailed personal data on smoking history, alcohol consumption, body mass index, physical activity level and dietary habits, so were therefore not adjusted for in this study and might be potential sources of bias.

## Conclusions

The findings of this population-based study show that the risk of major bleeding associated with combinations of CHMs and anticoagulants is lower than that with anticoagulants alone. Regardless, routine INR tests and clinical examinations are still needed to carefully monitor and assess bleeding risks in those patients using CHMs in combination with anticoagulant agents. Further studies are required to evaluate the benefits of the concomitant use of anticoagulants and specified CHMs as an integrative therapy.

## Supporting information

S1 File (DOCX)

S2 File Data of CHM cohort and non-CHM cohort in NHIRD. (XLSX)

## Abbreviations

aHR: adjusted hazard ratio
CAM: complementary and alternative medicine
CI: confidence interval
CHM: Chinese herbal medicine
ED: Emergency Department
ICD-9-CM: International Classification of Disease, Ninth Revision, Clinical Modification
LHID 2005: Longitudinal Health Insurance Database 2005
NHIRD: National Health Insurance Research Database
MI: myocardial infarction
PAD: peripheral arterial disease
TCM: traditional Chinese medicine
TE: thromboembolism
TIA: transient ischemic attack
