# Supplementary material for: Concurrent use of Chinese herbal medicine and anticoagulants may reduce major bleeding events
Source: PLoS One. 2022 Aug 23;17(8):e0271965. doi: 10.1371/journal.pone.0271965 (PMC9398017; doi:10.1371/journal.pone.0271965)
Supplement: S1 File — (DOCX) [file pone.0271965.s001.docx]

**Supplementary Table 1**

Medications of antiplatelet agents

| Other antiplatelet agents |
| --- |
| clopidogrel |
| ticlopidine |
| dipyridamole |
| iloprost |
| epoprostenol |
| tirofiban |
| treprostinil |
| cilostazol |

**Supplementary Table 2**

Compositions of CHM formulas

| Formula | Components |
| --- | --- |
| Shu Jing Huo Xue Tang. 疏經活血湯 | *Glycyrrhiza glabra* (Licorice Root, Gan Cao), *Wolfiporia extensa* (Hoelen, Fu Ling), *Gentianae Radix et Rhizoma* (Chinese Gentiana Root, Long Dan Cao), *Atractylodes lancea* (Black Atactylodis Rhizome, Cang Zhu), *Zingiber officinale* (Fresh Ginger Rhizome, Sheng Jiang), *Rehmannia glutinosa (Gaert.) Libosch. ex Fisch. et Mey.* (Rehmannia Root, Sheng Di Huang), *Angelicae Sinensis Radix* (Chinese Angelica Root, Dang Gui), *Saposhnikovia divaricata (Turcz.) Schischk.* (Saposhnikovia Root, Fang Feng), *Notopterygii Rhizoma et Radix* (Notopterygium Root, Qiang Huo), *Cyathula officinalis Kuan* (Cyanthula Root, Chuan Niu Xi), *Clematis chinensis Osbeck* (Clematis Root, Wei Ling Xian), *Stephaniae Tetrandrae Radix* (Stephania Root, Fen Fang Ji), *Paeonia lactiflora Pall* (White Peony Root, Bai Shao), *Prunus persica (L.) Batsch* (Peach Kernal, Tao Ren), *Ligusticum chuanxiong hort* (Cnidium, Chuan Xiong), *Angelica dahurica (Fisch. ex Hoffm.) Benth. et Hook. f. ex Franch. et Sav.* (Angelica Root, Bai Zhi), *Citri Reticulatae Pericarpium.* (Citrus Peel, Chen Pi). |
| Zhi Gan Cao Tang. 炙甘草湯 | *Jujubae Fructus* (Jujube Berry, Da Zao), *Asini Corii Colla* (Gelatin, E Jiao), *Zingiber officinale* (Fresh Ginger Rhizome, Sheng Jiang), *Ophiopogon japonicus (L.f.) Ker-Gawl.* (Dwarf Lilyturf Root, Mai Men Dong), *Rehmannia glutinosa (Gaert.) Libosch. ex Fisch. et Mey.* (Rehmannia Root, Sheng Di Huang), *Glycyrrhiza glabra* (Baked Licorice Root, Zhi Gan Cao), *Ginseng Radix et Rhizoma* (Ginseng Root, Ren Shen). *Cinnamomi Ramulus.* (Cinnamon Twig, Gui Zhi), *Cannabis sativa L.* (Linum, Huo Ma Ren). |
| Shao Yao Gan Cao Tang. 芍藥甘草湯 | *Paeonia lactiflora Pall* (White Peony Root, Bai Shao), *Glycyrrhiza glabra* (Licorice Root, Gan Cao). |
| Xue Fu Zhu Yu Tan. 血府逐瘀湯 | *Rehmannia glutinosa (Gaert.) Libosch. ex Fisch. et Mey.* (Rehmannia Root, Sheng Di Huang), *Angelicae Sinensis Radix* (Chinese Angelica Root, Dang Gui), *Cyathula officinalis Kuan* (Cyathula Root, Chuan Niu Xi), *Platycodon grandiflorum (Jacq.) A. DC.* (Platycodon Root, Jie Geng), *Paeonia anomala L. subsp. veitchii (Lynch) D. Y. Hong et K. Y. Pan* (Red Peony Root, Chi Shao), *Bupleuri Radix* (Bupleurum Root, Chai Hu), *Glycyrrhiza glabra* (Licorice Root, Gan Cao), *Prunus persica (L.) Batsch* (Peach Kernal, Tao Ren), *Citrus aurantium L.* (Bitter Orange, Zhi Qiao), *Ligusticum chuanxiong hort* (Cnidium, Chuan Xiong), *Carthamus tinctorius L.* (Safflower, Hong Hua). |
| Du Huo Ji Sheng Tan. 獨活寄生湯 | *Wolfiporia extensa* (Hoelen, Fu Ling), *Glycyrrhiza glabra* (Licorice Root, Gan Cao), *Taxillus chinensis (DC.) Danser* (Mulberry Mistletoe Stems, Sang Ji Sheng), *Gentiana macrophylla Pall. Fl. Ross.* (Gentiana Macrophylla Root, Qin Jiao), *Rehmannia glutinosa (Gaert.) Libosch. ex Fisch. et Mey.* (Rehmannia Root, Sheng Di Huang), *Angelica pubescens Maxim. F. biserrata Shan et Yuan* (Pubescent Angelica Root, Du Huo), *Angelicae Sinensis Radix* (Chinese Angelica Root, Dang Gui), *Saposhnikovia divaricata (Turcz.) Schischk.* (Saposhnikovia Root, Fang Feng), *Cinnamomi Ramulus.* (Cinnamon Inner Bark, Gui Xin), *Cyathula officinalis Kuan* (Cyathulae Root, Chuan Niu Xi), *Paeonia lactiflora Pall* (White Peony Root, Bai Shao), *Asarum sieboldii Miq.* (Chinese Wild Ginger, Xi Xin), *Eucommia ulmoides Oliver.* (Eucommia Bark, Du Zhong), *Ginseng Radix et Rhizoma* (Ginseng Root, Ren Shen), *Ligusticum chuanxiong hort* (Cnidium, Chuan Xiong). |
| Ji Sheng Shen Qi Wan. 濟生腎氣丸 | *Dioscorea opposita Thunb.* (Mountain Yam Rhizome, Shan Yao), *Paeonia suffruticosa Andrews* (Peony Root Bark, Mu Dan Pi Xi), *Cornus officinalis Sieb. et Zucc.* (Dogwood Fruit, Shan Zhu Yu), *Cyathula officinalis Kuan* (Cyathula Root, Chuan Niu Xi), *Plantago major Linn.* (Plantago Seed, Che Qian Zi), *Rehmannia glutinosa (Gaert.) Libosch. ex Fisch. et Mey.* (Prepared Rehmannia Root, Shou Di Huang), *Cinnamomum cassia Presl* (Cinnamon Bark, Rou Gui), *Alisma orientalis (Sam.) Juzep.* (Alisma Rhizome, Ze Xie), *Wolfiporia extensa* (Hoelen, Fu Ling). |
| Ge Gen Tang. 葛根湯 | *Zingiber officinale* (Fresh Ginger Rhizoma, Sheng Jiang), *Cimicifugae Rhizoma* (Bugbane Rhizome, Sheng Ma), *Paeonia lactiflora Pall* (White Peony Root, Bai Shao), *Glycyrrhiza glabra* (Licorice Root, Gan Cao), *Pueraria lobata (Willd.) Ohwi* (Kudzu Root, Ge Gen). |
| Sheng Mai Yin. 生脈飲 | *Ophiopogon japonicus (L.f.) Ker-Gawl.* (Dwarf Lily-Turf Root, Mai Men Dong), *Schisandra chinensis (Turcz.) Baill.* (Schizandra Fruit, Wu Wei Zi), *Ginseng Radix et Rhizoma* (Ginseng Root, Ren Shen). |
| Liu Wei Di Huang Wan. 六味地黃丸 | *Dioscorea opposita Thunb.* (Mountain Yam Rhizome, Shan Yao), *Paeonia suffruticosa Andrews* (Tree Peony Root Bark, Mu Dan Pi), *Cornus officinalis Sieb. et Zucc.* (Dogwood Fruit, Shan Zhu Yu), *Rehmannia glutinosa (Gaert.) Libosch. ex Fisch. et Mey.* (Prepared Rhemannia Root, Shou Di Huang), *Alisma orientalis (Sam.) Juzep.* (Alisma Rhizome, Ze Xie), *Wolfiporia extensa* (Hoelen, Fu Ling). |
| Ping Wei San. 平胃散(丸) | *Zingiber officinale* (Fresh Ginger Rhizome, Sheng Jiang), *Atractylodes lancea* (Black Atractylodis Rhizome, Cang Zhu), *Glycyrrhiza glabra* (Licorice Root, Gan Cao), *Magnolia officinalis Rehd. et E. H. Wils.* (Magnolia Bark, Hou Pu), *Jujubae Fructus* (Jujube Berry, Da Zao), *Citri Reticulatae Pericarpium.* (Citrus Peel, Chen Pi). |
